# Survival benefit of anti-angiogenic agents in patients with previously treated advanced gastric or gastroesophageal junction cancer: a meta-analysis

**DOI:** 10.18632/oncotarget.18314

**Published:** 2017-05-31

**Authors:** Jung Han Kim, Hyeong Su Kim, Bum Jun Kim, Hyun Joo Jang

**Affiliations:** ^1^ Division of Hemato-Oncology, Department of Internal Medicine, Kangnam Sacred-Heart Hospital, Hallym University Medical Center, Hallym University College of Medicine, Seoul 07441, Republic of Korea; ^2^ Division of Gastroenterology, Department of Internal Medicine, Dongtan Sacred-Heart Hospital, Hallym University Medical Center, Hallym University College of Medicine, Hwasung 18450, Republic of Korea

**Keywords:** gastric cancer, gastroesophageal junction cancer, anti-angiogenic agent, meta-analysis

## Abstract

There is a debate as to whether anti-angiogenic molecular agents can produce survival benefits in patients with previously treated advanced gastric cancer (GC) or gastroesophageal junction cancer (GEJC). We performed this meta-analysis of randomized trials to evaluate the survival outcomes of an anti-angiogenic agent versus placebo in the salvage treatment of advanced GC or GEJC. Electronic databases were searched for eligible studies. From the four studies, 910 patients with previously treated advanced GC or GEJC were included in the meta-analysis. Compared with placebo, anti-angiogenic targeted agents significantly improved progression-free survival (hazard ratio = 0.37 [95% confidence interval, 0.26–0.53], *P* < 0.00001). In terms of overall survival, anti-angiogenic agents induced 36% reduction in the risk for death (hazard ratio = 0.64 [95% confidence interval, 0.48–0.86], *P* = 0.002). In conclusion, this meta-analysis demonstrates that anti-angiogenic agents can prolong survival in patients with previously treated advanced GC or GEJC. This finding suggests that anti-angiogenic therapy can be a considerable option in patients who are not candidates for further chemotherapy.

## INTRODUCTION

Gastric cancer (GC) is one of the most common cancers worldwide, in terms of incidence as well as mortality [1, 2]. Although its incidence in individuals younger than 50 years has increased recently, GC develops more frequently among patients in their seventh or eighth decades [2]. Radical surgery with or without perioperative or adjuvant chemotherapy is the potential curative treatment for patients with localized GC, but a considerable number of patients present with advanced disease at the time of diagnosis. Moreover, more than half patients treated by complete resection eventually develop recurrent diseases during the course of their disease [3, 4]. Combination of fluoropyrimidine and platinum has been established worldwide as the first-line therapy for advanced GC [5]. However, most patients become resistant to the first-line chemotherapy. Single-agent irinotecan and taxane significantly improved overall survival (OS) compared with best supportive care in the second-line setting [6, 7], but unfortunately median OS was less than six months [8]. Thus, the development of more effective salvage treatment is still warranted.

Abnormal angiogenesis is well known as one of the main mechanisms of tumor growth and metastases [9]. Vascular endothelial growth factor (VEGF) and VEGF receptor-2 (VEGFR-2)-mediate signaling and angiogenesis contributes to the pathogenesis of GC [9, 10]. In preclinical studies, the inhibition of VEGF or VEGFR-2 signaling pathways has been shown to reduce tumor growth [11]. Recent clinical trials have found that single molecular agent targeting VEGFR-2 (ramucirumab or apatinib) as a salvage therapy may improve progression-free survival (PFS) and OS of patients with advanced GC or gastroesophageal junction cancer (GEJC) [12–14]. However, regorafenib, a dual targeted VEGFR2-TIE2 tyrosine kinase inhibitor (TKI), failed to show significantly longer OS than placebo in patients with refractory advanced GC or GEJC [15].

Until now, there is a debate as to whether anti-angiogenic molecular agents can produce survival benefit as monotherapy in patients with previously treated advanced GC or GEJC. We performed this meta-analysis of randomized trials to evaluate the survival outcomes of an anti-angiogenic agent versus placebo in the salvage treatment of advanced GC or GEJC

## RESULTS

### Results of search

**Figure 1:**
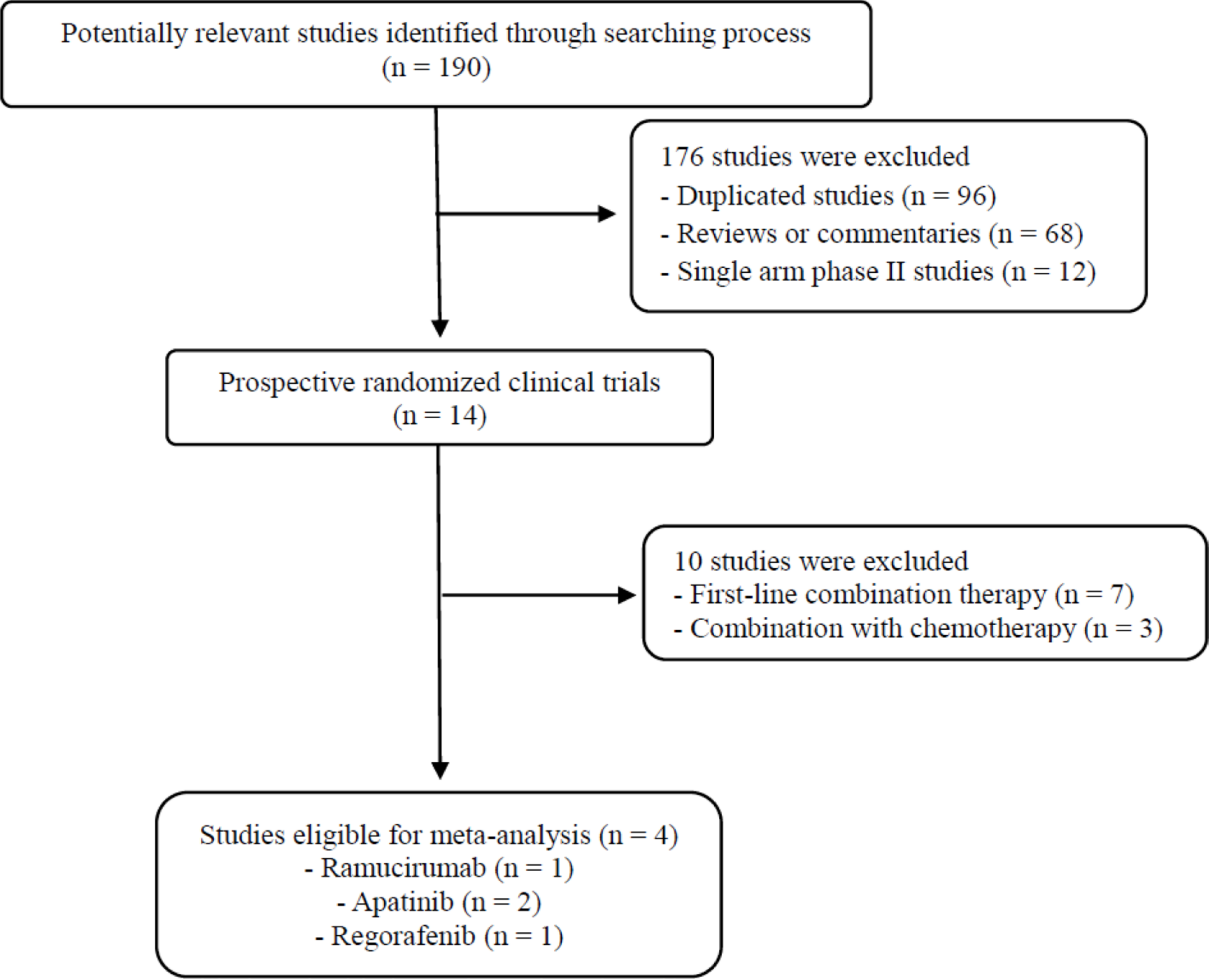
Flow diagram of search process

Figure 1 shows the flowchart of studies through the selection process. A total of 190 potentially relevant studies were initially yielded, but 176 of them were excluded by searching strategy after screening the titles and abstracts. Of the remaining 14 potentially relevant randomized studies, 10 were further excluded by the inclusion criteria; seven were conducted in the first-line treatment setting and two tested an anti-angiogenic agent in combination with chemotherapy. Finally, 4 randomized controlled phase II or III trials were included in the meta-analysis [12–15].

### Characteristics of the eligible studies

Table 1 summarizes the main characteristics and clinical outcomes of the four studies. The included studies reported outcomes with ramicirumab [12], apatinib [13, 14], and regorafenib [15]. A double-blind, placebo-controlled, phase II study by Li *et al.* randomized patients to receive apatinib 850 mg once daily, apatinib 425 mg twice daily, or placebo [13]. The overall response rates of the tested anti-angiogenic agents were 3 to 13% [12–14].

**Table 1:** Summary of the four eligible studies

Author (year) Study name	Phase/ Setting	Arms	Primary endpoint	No. of patients	ORR	Median PFS	HR for PFS (95% CI)	Median OS	HR for OS (95% CI)
Fuchs et *al.* (2014) REGARD	III/ 2nd-line	Ramucirumab Placebo	OS	238 117	3% 0%	2.1 1.4	0.48 (0.38–0.62) *P* < 0.001	5.2 3.8	0.78 (0.60–0.99) *P* = 0.047
Pavlakis *et al.* (2015) INTEGRATE	II/ 2nd- or 3rd-line	Regorafenib Placebo	PFS	97 50	NA NA	2.5 0.9	0.41 (0.28–0.59) *P* < 0.001	5.8 4.5	0.74 (0.51–1.08) *P* = 0.11
Li *et al.* (2013)	II/ 3rd-line or further	Apatinib 850 mg Apatinib 425 mg Placebo	PFS	47 46 48	13% 6% 0%	3.7 3.2 1.4	0.18 (0.10–0.34) *P* < 0.001 0.21 (0.11–0.38) *P* < 0.001	4.8 4.3 2.5	0.37 (0.22–0.62 *P* < 0.001 0.41 (0.24–0.71) *P* < 0.001
Li *et al.* (2016)	III/ 3rd-line or further	Apatinib Placebo	PFS & OS	176 91	3% 0%	2.6 1.8	0.44 (0.33–0.60) *P* < 0.001	6.5 4.7	0.71 (0.54–0.94) *P* = 0.0156

PFS, progression-free survival; OS, overall survival; HR, hazard ratio; CI, confidence interval; ORR, overall response rate; NA, not available.

### Survival analyses

**Figure 2:**
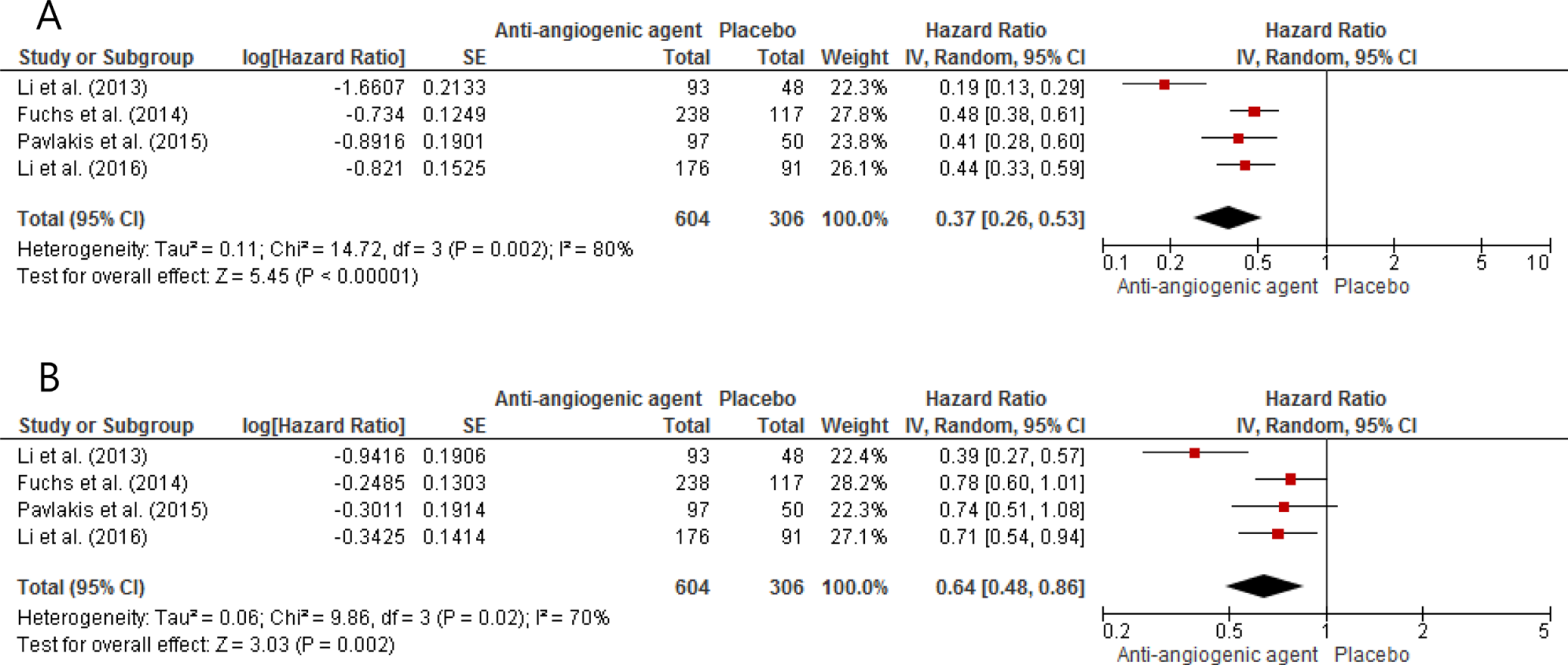
Forest plots of hazard ratios regarding progression-free survival (**A**) and overall survival (**B**) of anti-angiogenic agents versus placebo.

From the four studies, 910 patients were included in the meta-analyses of hazard ratio (HR) for PFS and OS. Compared with placebo, anti-angiogenic targeted agents significantly improved PFS (HR = 0.37 [95% confidence interval (CI), 0.26–0.53], *P* < 0.00001) (Figure 2A). We adopted the random-effect model because there was significant heterogeneity (*X*^2^ = 14.72, *P* = 0.002, *I*^2^ = 80%).

In terms of OS, anti-angiogenic agents induced 36% reduction in the risk for death (HR = 0.64 [95% CI, 0.48–0.86], *P* = 0.002), compared with placebo (Figure 2B). The random-effect model was used because there was significant heterogeneity (*X*^2^ = 9.86, *P* = 0.02, *I*^2^ = 70%).

## DISCUSSION

We performed this study to investigate the survival benefits of anti-angiogenic agents as a rescue therapy after failure of previous chemotherapy in patients with advanced GC or GEJC. The meta-analysis of four randomized studies indicates that anti-angiogenic therapy alone can prolong survival over that with placebo in patients with previously treated advanced GC or GEJC.

Although several angiogenesis inhibitors have proved their efficacy in patients with renal carcinoma, hepatocellular carcinoma, or colorectal cancer, they have shown limited clinical value in advanced GC. Especially, the AVAGAST study evaluating the efficacy of bevacizumab, a monoclonal antibody to VEGF, in combination with chemotherapy in patients with chemotherapy-naïve advanced GC failed to show statistically significant improvement of PFS and OS [16]. However, a growing number of molecular targeted agents with anti-angiogenic effects have been evaluated in various setting of metastatic GC or GEJC.

There are several randomized clinical trials that have tested an anti-angiogenic agent as a rescue therapy in comparison with placebo in patients with chemo-refractory advanced GC or GEJC. The REGARD study evaluated the efficacy of ramucirumab, a monoclonal antibody VEGFR-2 antagonist, versus placebo in the second-line treatment of advanced GC or GEJC [12]. Patients treated with ramucirumab showed significantly prolonged PFS and OS compared with those who received placebo. Importantly none of adverse events associated with ramucirumab reached statistical significance compared with placebo. Li *et al.* performed a randomized phase II trial with apatinib, a small molecule TKI selectively targeting VEGFR-2, in 144 patients with advanced GC who failed at least two chemotherapeutic regimens [13]. The enrolled patients were randomized to receive placebo, 850 mg apatinib once daily, or 450 mg apatinib twice daily. Patients in the apatinib groups achieved significantly improved PFS and OS, compared with those in the placebo group. Then they conducted a phase III trial of treating chemo-refractory advanced GC or GEJC with apatinib (850 mg once daily) [14]. Patients treated with apatinib showed significantly longer PFS and OS than those who received placebo. In terms of adverse events, apatinib was associated with the increased incidence of grade 3–4 hand-foot syndrome (9% vs. 1%) and hypertension (6% vs. 0%) [17]. Regorafenib had been also tested in a randomized, placebo-controlled phase II study of patients with refractory advanced GC or GEJC [15]. Regorafenib group showed significantly longer PFS than placebo group with no significant increase of severe toxicities, but OS improvement of regorafenib showed only a positive trend (5.8 vs. 4.5 months, HR = 0.74, *P* = 0.11).

In this meta-analysis of those four randomized, placebo-controlled trials with previously treated advanced GC or GEJC, anti-angiogenic targeted agents significantly improved both PFS (HR = 0.37, *P* < 0.00001) and OS (HR = 0.64, *P* = 0.002) compared with placebo. Considering favorable toxicity profile observed in the individual studies [12–15], this finding suggests that anti-angiogenic agents can be considered a valuable therapeutic option in patients who cannot receive further chemotherapy.

To date, no biomarkers are available to predict the benefit of anti-angiogenic agents in the treatment of solid tumors. The pre-planned correlative analyses of the AVAGAST trial identified baseline VEGF-A level and tumor neuropilin-1 expression as potential predictors of bevacizumab efficacy [18]. Patients with high baseline plasma VEGF-A levels showed a trend toward improved OS (HR = 0.72, 95% CI, 0.57–0.93), compared with those with low VEGF-A levels (HR = 1.01 [95% CI, 0.77–1.31], interaction *P* = 0.07). Patients with low baseline expression of neuropilin-1 also showed a trend toward improved OS (HR = 0.75 [95% CI, 0.59–0.97]) versus those with high neuropilin-1 expression (HR = 1.07 [95% CI, 0.81–1.40], interaction *P* = 0.06). For both biomarkers, however, subgroup analyses showed significance only in non-Asian patients. Therefore, there is a critical need to discover new biomarkers that can predict the efficacy of these anti-angiogenic agents.

The current study has inherent limitations that should be noted. This study included the small number of trials currently available. The individual studies had been conducted in different treatment settings with different anti-angiogenic agents. In addition, the impact of anti-angiogenic therapy on quality of life was not analyzed due to the lack of available data. In the REGARD study, however, patients in the ramucirumab group more frequently reported stable or improved EORTC QLQ-C30 global health score than those in the placebo group, although this difference was not significant (*P* = 0.21) [12].

In conclusion, this meta-analysis demonstrates that anti-angiogenic agents can prolong survival in patients with previously treated advanced GC or GEJC. This finding suggests that anti-angiogenic therapy can be a considerable option in patients who are not candidates for further chemotherapy.

## MATERIALS AND METHODS

### Searching strategy

This study was carried out according to the Preferred Reporting Items for Systematic Reviews and Meta-Analyses (PRISMA) guidelines [19]. We performed a systematic search of electronic databases, such as PubMed, Embase, and the Cochrane Central Register of Controlled Trials (CENTRAL) up to December 2016. We also manually searched the following congress abstract databases: American Society for Clinical Oncology (ASCO) Annual Meeting, ASCO Gastrointestinal Cancers Symposium, and European Society for Medical Oncology (ESMO) Congress. Prospective studies were only allowed to reduce the risk of selection bias. The search was performed using the following keywords: ‘stomach or gastric cancer’, ‘gastroesophageal junction cancer’, ‘randomized’, ‘anti-angiogenic agent or anti-angiogenic therapy’, ‘vascular endothelial growth factor inhibitor or VEGF inhibitor’, ‘bevacizumab’, ‘ramucirumab’, ‘apatinib’, ‘regorafenib’, ‘sunitinib’, ‘sorafenib’, ‘pazopanib’, ‘orantinib’, ‘telatinib’ in various combinations. The related articles function in the PubMed was also used to identify all related articles.

### Inclusion criteria

Eligible studies were required to meet the following inclusion criteria: prospective randomized, controlled trials in patients with previously treated advanced GC or GEJC; randomization of patients to either anti-angiogenic therapy or best supportive care with or without placebo; providing HRs and their 95% CIs for PFS or OS.

### Data extraction

Data extraction was carried out independently by two investigators (BJK and JHK). If these two authors did not agree, the other investigators (HSK or HJJ) were consulted to resolve the dispute. The following data were extracted from all eligible studies: first author’s name, year of publication, trial phase and setting, number of enrolled patients, the used anti-angiogenic agent, overall response rates, PFS or OS with their HRs and 95% CIs.

### Statistical analysis

Statistical values used in this meta-analysis were obtained directly from the original article. Heterogeneity among studies was estimated using the *I*^2^ inconsistency test and chi-square-based Cochran’s *Q* statistic test in which *P* < 0.1 was taken to indicate the presence of significant heterogeneity. The fixed-effect model (Mantel-Haenszel method) was used to calculate the pooled HR when substantial heterogeneity was not detected. When substantial heterogeneity was observed, we adopted the random-effects model (DerSimonian-Laird method). The RevMan software version 5.2 was used to report outcomes, and the final results were presented with HR and 95% CI. All reported *P*-values were two-sided and *P* < 0.05 was considered statistically significant.
